# Supplementary material for: Two distinct superconducting states controlled by orientations of local wrinkles in LiFeAs
Source: Nat Commun. 2021 Nov 2;12:6312. doi: 10.1038/s41467-021-26708-8 (PMC8563765; doi:10.1038/s41467-021-26708-8)
Supplement: Supplementary file 1 — Supplementary Information [file 41467_2021_26708_MOESM1_ESM.pdf]

Supplemental Information for

Two distinct superconducting states controlled by orientations of local wrinkles in LiFeAs

Lu Cao[1,2,5], Wenyao Liu[1,2,5], Geng Li[1,2,3,4,5*], Guangyang Dai[1,2], Qi Zheng[1,2], Yuxin Wang[1,2], Kun Jiang[1,2], Shiyu Zhu[1,2], Li Huang[1,2,3], Lingyuan Kong[1], Fazhi Yang[1,2], Xiancheng Wang[1,3,4], Wu Zhou[2,3], Xiao Lin[2,1], Jiangping Hu[1,2,3], Changqing Jin[1,3,4], Hong Ding[1,3,4*], Hong-Jun Gao[1,2,3,4*]

[1]Institute of Physics, Chinese Academy of Sciences, Beijing 100190, China

[2]School of Physical Sciences, University of Chinese Academy of Sciences, Beijing 100049, China

[3]CAS Center for Excellence in Topological Quantum Computation, University of Chinese Academy of Sciences, Beijing 100190, China

[4]Songshan Lake Materials Laboratory, Dongguan, Guangdong 523808, China

[5]These authors contributed equally: Lu Cao, Wenyao Liu, Geng Li

[*]Correspondence to: hjgao@iphy.ac.cn, dingh@iphy.ac.cn,

gengli.iop@iphy.ac.cn

Supplementary Note 1: The lattice deformation of wrinkles

The lattice deformation at the wrinkle location is negligible from the real-space atomic resolution image (Supplementary Fig. 1a). In the reciprocal space, FFT shows sharp first-order Bragg points marked by red and orange circles (Supplementary Fig. 1b). By extracting the line-profile of the first-order Bragg points in k_x and k_y directions (Supplementary Figs. 1c-1d), it can be calculated the lattice constant a_x = 3.831 Å for k_x direction and a_y = 3.846 Å for k_y direction. The difference is only 0.015 Å (or 0.4 %). We note that the value of 0.4 % is with large error bar since the effects such as drifting or piezo calibration could contribute to this small value. Nevertheless, it is still much smaller than the STM resolution limit (~0.1 Å). Therefore, by STM, we cannot extract the precise lattice deformations.

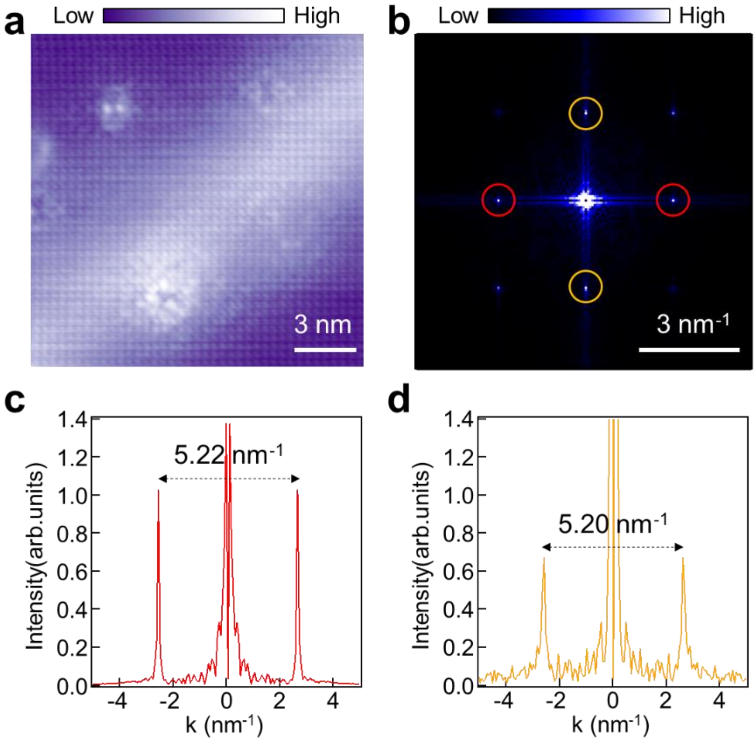

Supplementary Figure 1. The lattice constant of wrinkle. a, Atomic resolution STM image of type-I wrinkle, this is the same shown in Fig. 1**e**. **b**, The corresponding FFT of **a**. The red and orange circles marked the first-order Bragg points. **c**-**d**, the line-profile of Bragg points in k_x (red) and k_y (orange) directions, respective.

Supplementary Note 2: The real topography of wrinkles

In STM, it is well known that the tunneling current is proportional to the integral of the density of states (DOS) from 0 to the setpoint bias. So the topography obtained from the constant-current mode is the contour of the constant DOS, which strictly speaking is not the real topography. To clarify the wrinkle structure observed by STM has a real hump on topography rather than a result from the high DOS accumulation or charge accumulation, we carry out the atomic force microscopy (AFM) measurements. Unlike the STM, AFM acquires the atomic force between the tip and the sample, thus can be used to detect the real topography. We first use the constant-current STM mode to find the type-I and II wrinkles as well as the wrinkle-free region (Supplementary Figs. 2a-2b). Then, the constant-high AFM maps are used to detect the atomic force on the wrinkles and wrinkle-free region (Supplementary Figs. 2c-2d). Note that the stronger atomic force (dark color in Supplementary Figs. 2c-2d) means the shorter tip-sample distance, therefore the wrinkles have a real hump topography due to the constant-high mode. We also use the constant-force AFM mode to check the wrinkles (Supplementary Figs. 2e-2f). In the constant-force mode, the tip-sample distance is tuned to keep the atomic force keep constant. So the absolute height of topography can be detected. The results of the constant-force line profile shown in Supplementary Figs. 2e-2f demonstrate again that the wrinkles have a real hump topography.

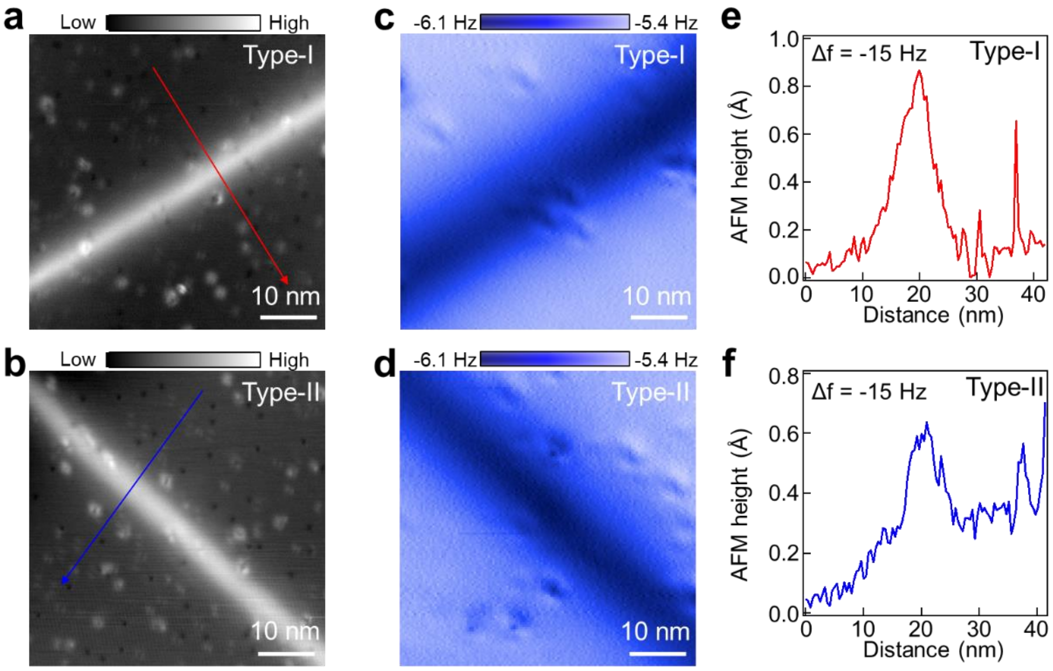

Supplementary Figure 2. Atomic force microscopy (AFM) measurements on wrinkles. a-b, STM topographies of type-I and type-II wrinkles, respectively. **c-d**, Constant-height AFM maps on the two types of wrinkles. The bright and dark color scale correspond to the weak and strong tip-sample interactions, respectively, indicating that the two types of wrinkles have real spatial corrugations on the LiFeAs surface. **e-f**, Constant-force AFM line-profile measured along the arrows marked in **a** and **b**.

Supplementary Note 3: Gap map at type-I wrinkle

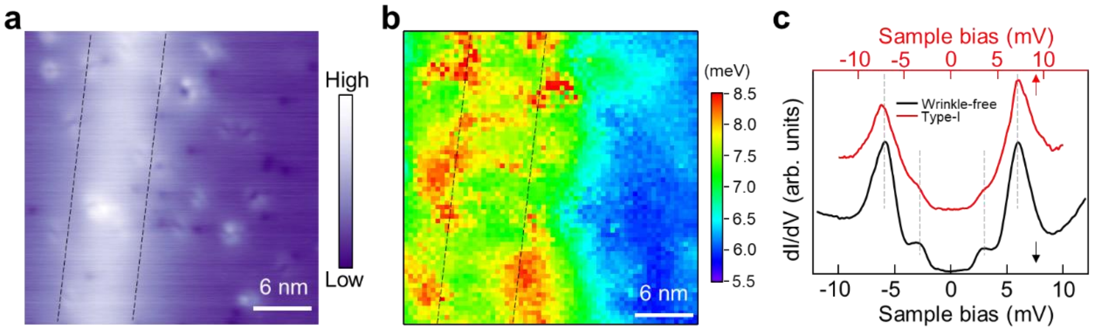

Supplementary Figure 3. Gap map at type-I wrinkle. a, A zoom-in STM topography of a type-I wrinkle. Black dashed lines indicate the edges of the wrinkle. **b**, A gap map of Δ_1 taken at a type-I wrinkle. **c**, Comparison of the SC gaps between the wrinkle-free region and type-I region in different axis scales. The grey dashed lines mark the two coherence peaks. For type-I region, the two gaps (Δ_1 and Δ_2) increase at an equal ratio.

Supplementary Note 4: The vortex lattice on wrinkles

Although the type-I and II wrinkles have different orientations, the vortices on wrinkles present a lattice structure with equal distance (Supplementary Figs. 4b and 4d). More importantly, compared with the vortex shape on wrinkle-free regions, they all break the C_4 symmetry with an oval-like shape with the long axis along the wrinkles, which strongly indicates not only that the local strain exists at the wrinkles, but also that the local electronic state is dramatically changed.

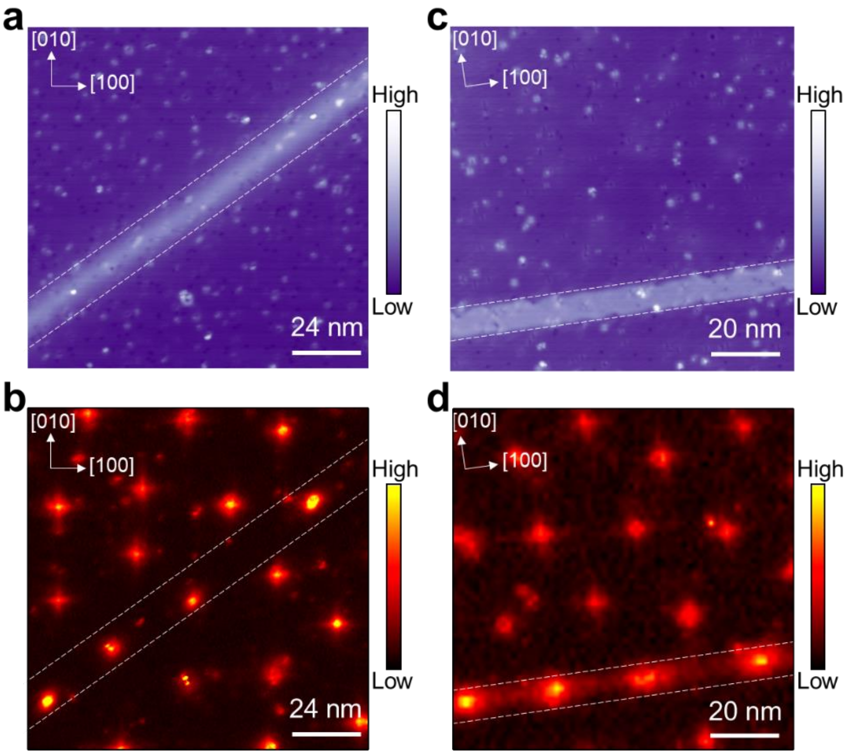

Supplementary Figure 4. Comparison of vortices on wrinkle-free region and wrinkle regions. a, A large scale STM topography image with wrinkle-free region and type-I wrinkle region included. White dashed lines mark the edge of wrinkle. **b**, The zero-bias conductance (ZBC) map of the same area in **a** under a magnetic field of 3 T, showing the vortices distributed in the whole area. **c**-**d**, Same as **a** and **b**, but with type-II wrinkle region included and under the magnetic field of 4 T. The [100] and [010] mark the lattice directions on the Li terminal surface, respectively.

Supplementary Note 5: The transition between type-I and type-II wrinkles

We find a kink that divide the type-I and type-II wrinkles (Supplementary Fig. 5a), and the dI/dV spectra from the type-II wrinkles to type-I wrinkles (Supplementary Fig. 5b) exhibit the SC gaps transition from a single gap feature with 2.6 meV to the two enlarged gap features with 7.0 meV and 4.0 meV.

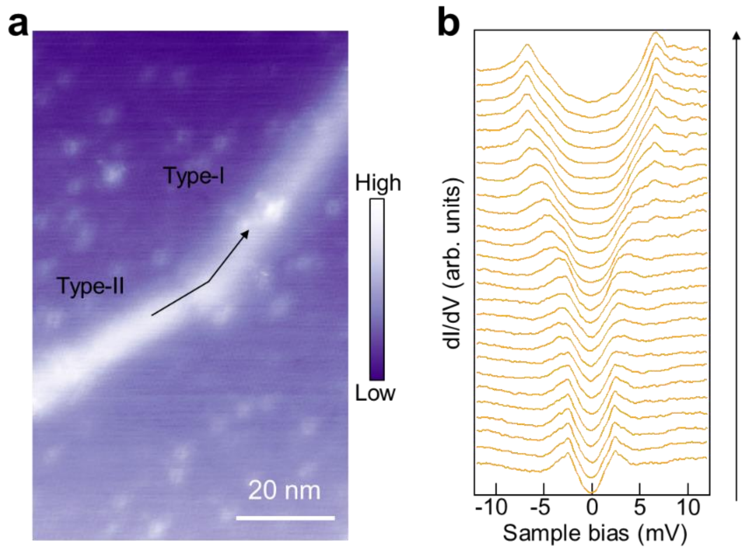

Supplementary Figure 5. Transition between type-I and type-II wrinkles. a, A STM topography showing the kink which divides the type-I and type-II wrinkles. **b**, The dI/dV spectra taken along the arrows marked in a, showing the SC gap transition from type-II to type-I wrinkles.

Supplementary Note 6: The wide range d*I*/d*V* spectra on wrinkles

The hump feature at ~30 meV corresponds to the band top of the d_{xy} band, which shows a slight shift at different regions. The second differential curves (Supplementary Fig. 6a) of the data further indicates the local band (d_{xy}) shift on the wrinkles. We find that the d_{xy} band shifts upward at type-I region and downward at type-II region.

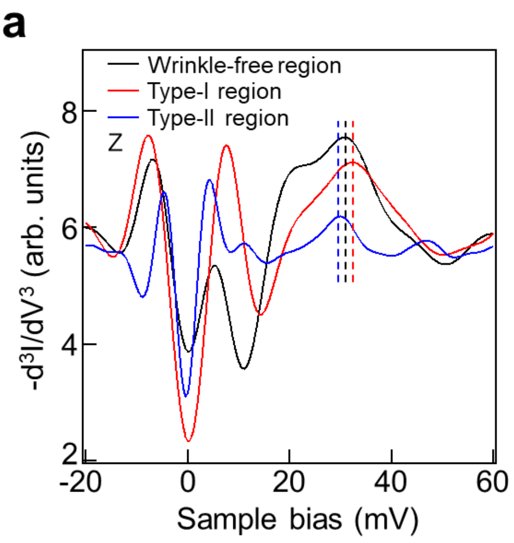

Supplementary Figure 6. The negative of 2nd differential curves of wide range *dI/dV* spectra. a, The negative of 2nd differential curves of the spectra in Fig. 4(a). The dashed lines indicate the energy positions of the d_{xy} band top.

Supplementary Note 7: Correlation of gap sizes and wrinkle width.

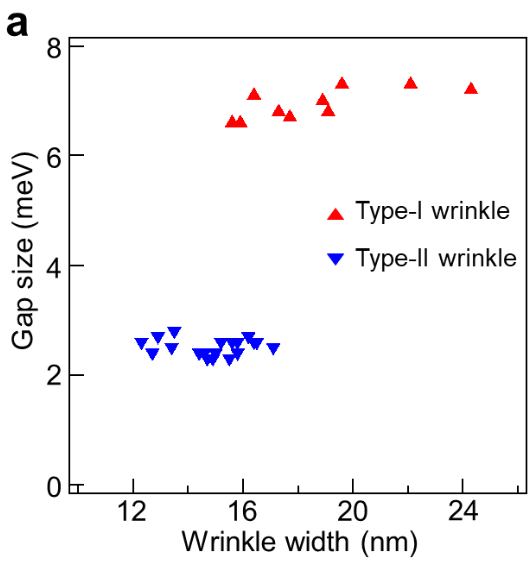

Supplementary Figure 7. The statistics of gap sizes versus wrinkle width. a, The statistics of the gap size versus wrinkle width for type-I and type-II wrinkles. The averaged width for type-I is large than type-II. For each bunch of type-I and type-II wrinkles, the width has no correlation with the gap size.

Supplementary Note 8: DFT calculations.

DFT calculations are conducted to verify the local strain effects on band shifts. We note that simulation of the complete wrinkle structure is unrealistic due to the requirement of large computational resources. Therefore, we constructed a simplified model to evaluate the strain effects along Fe-Fe and Fe-As directions by intentionally changing the distance between two adjacent rows of Fe-Fe atoms or Fe-As atoms (See method and Supplementary Fig. 8a-8b). Under Fe-Fe direction strain, the results show that the d_{xz} band shift up (down) under the tensile (compressive) strain (red arrows in Supplementary Fig. 8c-8e). However, when subjected to Fe-As direction strain, the d_{xz} band top shift up (down) under the compressive (tensile) strain (blue arrows in Supplementary Fig. 8f-8h). In solids, wrinkles possess both tensile and compressive strain[1,2]. Typically, the tensile strain is expected at the ridge region while the compressive strain exists at the valley region[1,2]. The band shifts induced by tensile strain agree with the proposed Lifshitz transition scenario in Fig. 4e-4g. Thus, we conjecture that the tensile strain dominates the electronic behavior of the wrinkle areas.

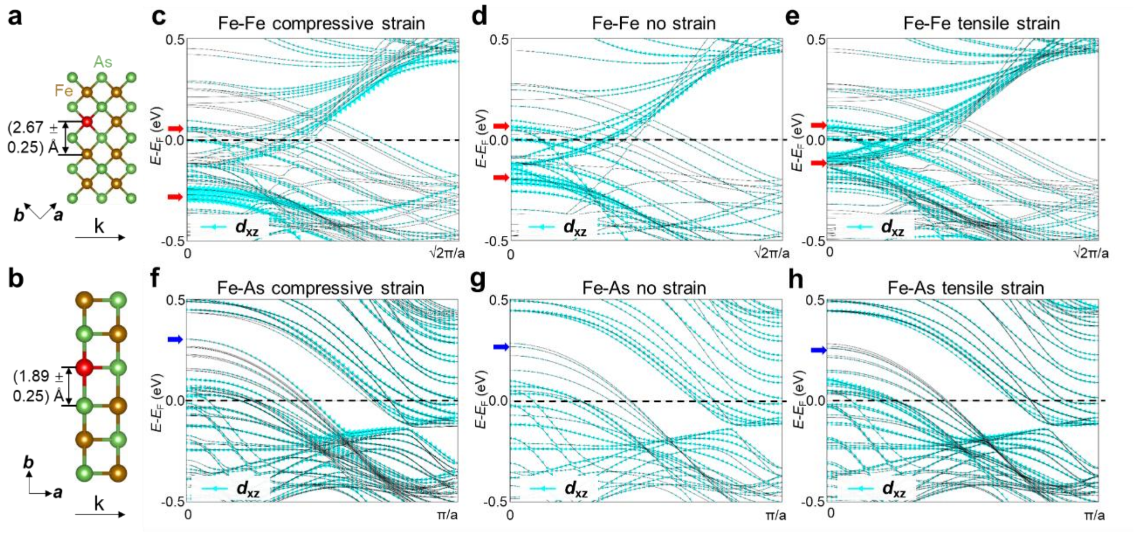

Supplementary Figure 8. DFT calculations under strain. **a**, The sketch for DFT calculations supercell model under Fe-Fe direction strain. The real supercell contains 40 Li atoms, 40 Fe atoms and 40 As atoms. We choose the Fe-Fe bonds at the supercell center and increase (decrease) their bond lengths 0.25 Å to account for the tensile (compressive) strain, while keeping other bonds unchanged. The x-axis of the Cartesian coordinate system is set along the Fe-Fe direction. The orbital projected band structures are calculated via this supercell model. The red atom is the Fe from which the d_{xz} band is extracted. The arrow indicates the periodic direction of supercell. The plane-wave cut-off energy is 600 eV. In self-consistent calculations, k point is $13 \times 1 \times 8$ with Γ centered. The energy convergence accuracy is 10^{-8} eV. **b**, Same with **a** but under Fe-As direction strain. And the energy convergence accuracy is 10^{-6} eV. **c-e**, The calculated band structure of the supercell described in **a** under compressive strain, no strain and tensile strain along Fe-Fe direction, respectively. The red arrows highlight the energy positions of the d_{xz} band near the Γ point. **f-h**, Same with **a-c** but along Fe-As direction. The blue arrows highlight the energy position of the d_{xz} band top near Γ point.

Supplementary Note 9: Type-I wrinkles along different orientations.

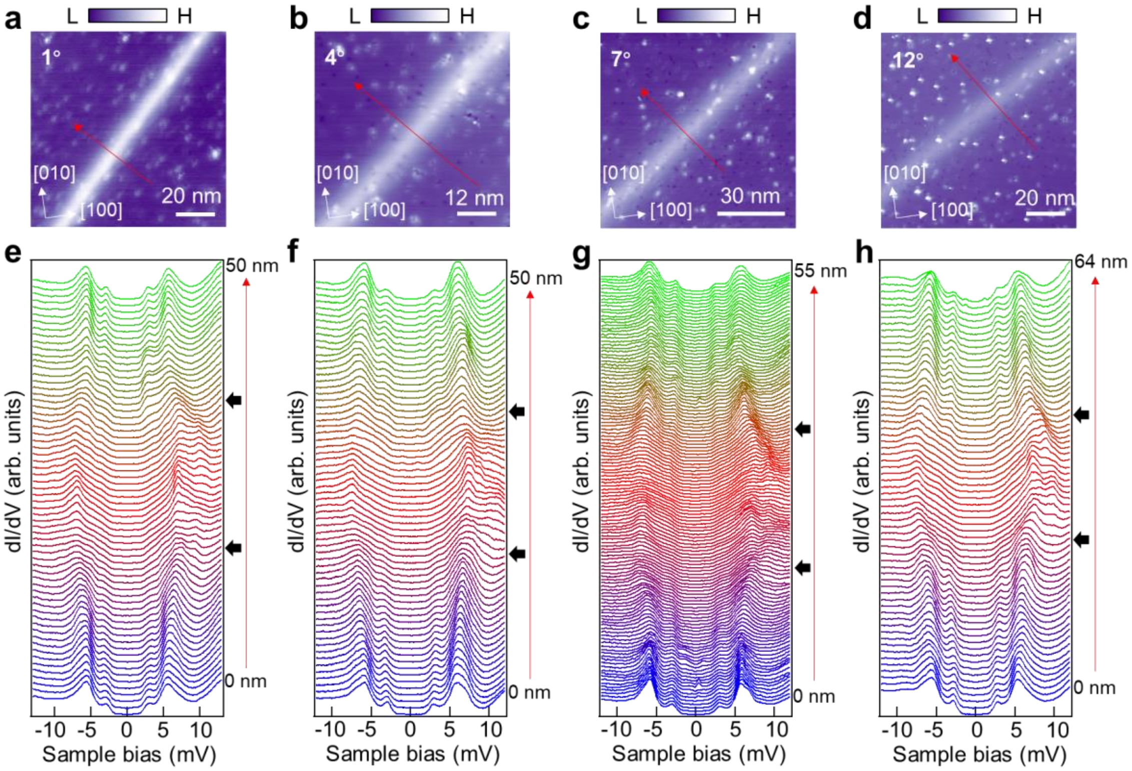

Supplementary Figure 9. Type-I wrinkles along different orientations. a-d, STM topography images for type-I wrinkles along different orientations. The angle between the wrinkles' orientations and the [110] crystal direction are marked in the upper left corner. **e-h**, The corresponding dI/dV spectra line-cut taken along the solid arrows in **a-d**, respectively. Enhanced gap emerges at the location of type-I wrinkle. Black arrows indicate the wrinkle boundary.

Supplementary Note 10: Type-II wrinkles along different orientations.

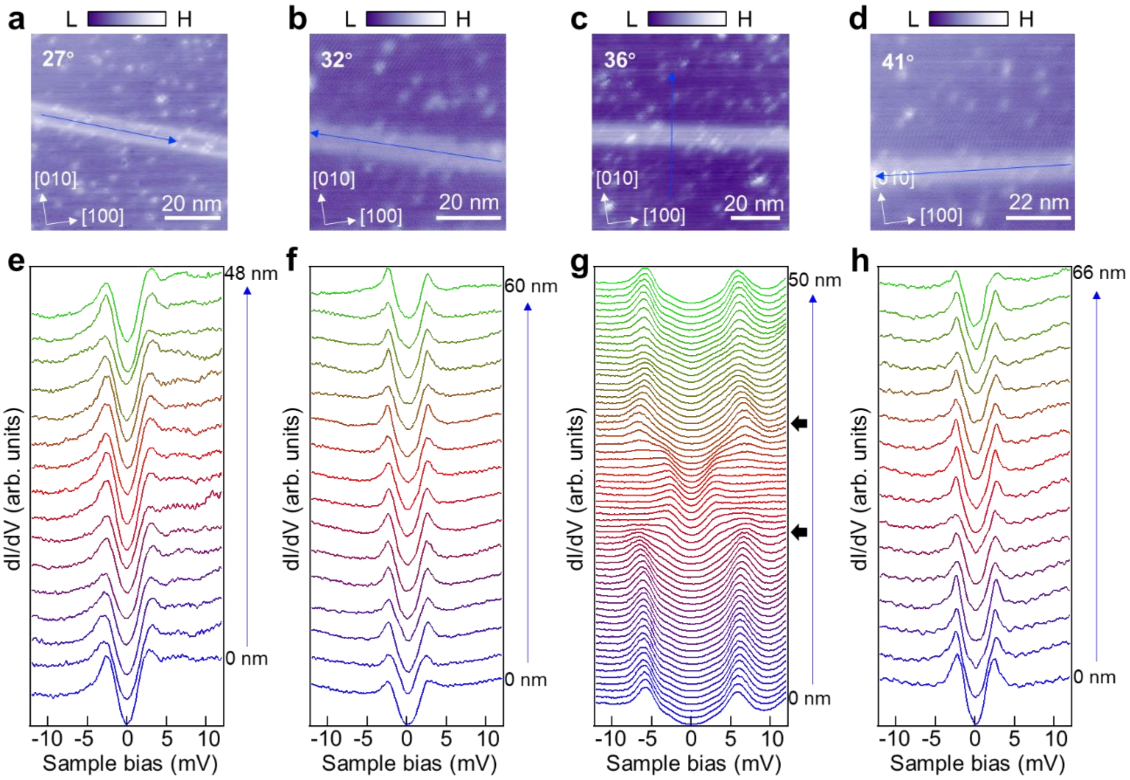

Supplementary Figure 10. Type-II wrinkles along different orientations. a-d, STM topography images for type-II wrinkles along different orientations. The angle between the wrinkles' orientations and the [110] crystal direction are marked in the upper left corner. **e-h**, The corresponding dI/dV spectra line-cut taken along the solid arrows in **a-d**, respectively. Suppressed gap emerges at the location of type-II wrinkle. Black arrows indicate the wrinkle boundary.

Supplementary Note 11: Fitting method.

The superconducting gaps are extracted by fitting the dI/dV spectra. First, the dI/dV below T_c are normalized by dividing the normal state dI/dV (T>T_c) to remove the background. Next, we fit the normalized dI/dV curve using the Dynes formula as following:

$$\frac{\mathrm{d}I}{\mathrm{d}V} \propto \int\int_0^{2\pi} \frac{df(E+eV,T)}{dV} N(E) d\theta_k dE$$

where

$$N(E) = \mathrm{Re}(\frac{E-\mathrm{i}\Gamma}{\sqrt{(E-\mathrm{i}\Gamma)^2 - \Delta(\theta_k)^2}})$$

and the $f(E + eV, T)$ is the Fermi-Dirac distribution function at the temperature T. E is the energy, Γ the inverse of quasiparticle lifetime, and $\Delta(\theta_k)$ the gap function in k-space. The temperature broadening effect is included in the Fermi-Dirac distribution function. According to the ARPES results, the $\Delta(\theta_k)$ for LiFeAs exhibit a 4-fold symmetry. So we adopt the 4-fold symmetry gap function for the gaps. As previously reported[3], in the fitting of gaps of the type-I wrinkle region and wrinkle-free region, the total dI/dV spectra value is contributed by the two gaps Δ_1 and Δ_2: dI/dV = PdI/dV (Δ_1) + (1-P)dI/dV (Δ_2). P is the weight and we adopt $P = 0.7$ in our fitting. And $\Delta_1 = \Delta_a(1 + 0.1\cos(4\theta_k))$, $\Delta_2 = \Delta_b(1 + 0.1\cos(4\theta_k))$. In the fitting of gap of the type-II wrinkle region, only Δ_2 are used and $\Delta_2 = \Delta_b(0.1 + \cos(4\theta_k))$.

Supplementary Note 12: Summary of gap size, T_c and $2\Delta/k_BT_c$.

	Wrinkle free		Type-I wrinkle		Type-II wrinkle
	Δ_1	Δ_2	Δ_1	Δ_2	Δ_2
Gap size	5.8	2.9	7.3	3.6	2.5
T_c	17		20.5		17
$2\Delta/k_BT_c$	7.9	4.0	8.3	4.1	3.4

Supplementary Table 1. Summary of gap size, T_c and $2\Delta/k_BT_c$.

References

1. Xu, L. *et al.* In-situ TEM investigation of MoS_2 wrinkles and its effects on electrical properties. *Mater. Chem. Phys.* **257**, 123797 (2021).
2. Castellanos-Gomez, A. *et al.* Local strain engineering in atomically thin MoS_2. *Nano Lett.* **13**, 5361-6 (2013).
3. Shan, L. *et al.* Observation of ordered vortices with Andreev bound states in $Ba_{0.6}K_{0.4}Fe_2As_2$. *Nat. Phys.* **7**, 325-331 (2011).
